# Upregulation of mitotic bookmarking factors during enhanced proliferation of human stromal cells in human platelet lysate

**DOI:** 10.1186/s12967-019-02183-0

**Published:** 2019-12-30

**Authors:** Sandra Laner-Plamberger, Michaela Oeller, Cornelia Mrazek, Arnulf Hartl, Alina Sonderegger, Eva Rohde, Dirk Strunk, Katharina Schallmoser

**Affiliations:** 1 Department of Transfusion Medicine, University Hospital of Salzburg (SALK), Paracelsus Medical University, Salzburg, Austria; 2 Spinal Cord Injury and Tissue Regeneration Center Salzburg (SCI-TReCS), Paracelsus Medical University, Strubergasse 21, 5020 Salzburg, Austria; 3 Department of Laboratory Medicine, University Hospital of Salzburg (SALK), Paracelsus Medical University, Salzburg, Austria; 4 Institute of Ecomedicine, Paracelsus Medical University, Salzburg, Austria; 5 Cell Therapy Institute, Paracelsus Medical University, Salzburg, Austria

**Keywords:** Stromal cells, Pooled human platelet lysate (pHPL), Fibrinogen, Fetal bovine serum (FBS), Mitotic bookmarking/transcription factors

## Abstract

**Background:**

Innovative human stromal cell therapeutics require xeno-free culture conditions. Various formulations of human platelet lysate (HPL) are efficient alternatives for fetal bovine serum (FBS). However, a consistent lack of standardized manufacturing protocols and quality criteria hampers comparability of HPL-products. Aim of this study was to compare the biochemical composition of three differential HPL-preparations with FBS and to investigate their impact on stromal cell biology.

**Methods:**

Stromal cells were isolated from bone marrow (BM), white adipose tissue (WAT) and umbilical cord (UC) and cultured in medium supplemented with pooled HPL (pHPL), fibrinogen-depleted serum-converted pHPL (pHPLS), mechanically fibrinogen-depleted pHPL (mcpHPL) and FBS. Biochemical parameters were analyzed in comparison to standard values in whole blood. Distinct growth factors and cytokines were measured by bead-based multiplex technology. Flow cytometry of stromal cell immunophenotype, in vitro differentiation, and mRNA expression analysis of transcription factors SOX2, KLF4, cMYC, OCT4 and NANOG were performed.

**Results:**

Biochemical parameters were comparable in all pHPL preparations, but to some extent different to FBS. Total protein, glucose, cholesterol and Na^+^ were elevated in pHPL preparations, K^+^ and Fe^3+^ levels were higher in FBS. Compared to FBS, pHPL-based media significantly enhanced stromal cell propagation. Characteristic immunophenotype and in vitro differentiation potential were maintained in all four culture conditions. The analysis of growth factors and cytokines revealed distinct levels depending on the pre-existence in pHPL, consumption or secretion by the stromal cells. Interestingly, mRNA expression of the transcription and mitotic bookmarking factors cMYC and KLF4 was significantly enhanced in a source dependent manner in stromal cells cultured in pHPL- compared to FBS-supplemented media. SOX2 mRNA expression of all stromal cell types was increased in all pHPL culture conditions.

**Conclusion:**

All pHPL-supplemented media equally supported proliferation of WAT- and UC-derived stromal cells significantly better than FBS. Mitotic bookmarking factors, known to enable a quick re-entry to the cell cycle, were significantly enhanced in pHPL-expanded cells. Our results support a better characterization and standardization of humanized culture media for stromal cell-based medicinal products.

## Background

The therapeutic potential of human so-called ‘mesenchymal’ stromal cells (‘MSCs’) is currently tested in more than 700 registered studies (*clinicaltrials.gov*), mainly targeting bone and cartilage regeneration, autoimmune diseases, cardiovascular and neurological disorders [1]. The stem-like identity of ‘MSCs’ used in these trials is still a matter of debate. A recent discussion by Robey [2] about origin, identity and terminology of ‘mesenchymal stem/progenitor cells’ has prompted us to use the more accurate term of tissue-derived ‘stromal cells’. Irrespective of the nomenclature, the clinical benefit of these cells based on paracrine and immunomodulatory effects is not yet sufficiently proven and many aspects such as culture conditions may affect their properties [3].

For manufacturing cell-based medicinal products fetal bovine serum (FBS) is still frequently used as medium supplement, bearing the risk of transmission of bovine pathogens and xeno-immunization [4, 5]. Also ethical issues concerning the manufacturing process of FBS have to be considered [5–7] and in 2007 the European Medicine Agency (EMA) has discouraged the use of animal-derived raw materials for manufacturing of cell therapeutics [8]. The efficient use of human platelet lysate (HPL) for the expansion of stromal cells was introduced in 2005 [9] and confirmed by us and others [6, 10–12]. Containing abundant growth factors, cytokines and plasma proteins such as thrombin and fibrinogen [13–15], HPL is used for isolation and large scale expansion of stromal cells from different tissues [10, 11, 16–18].

Various preparation techniques may differentially affect the composition of HPL [6], potentially influencing the biological properties of cultured cells [15, 19]. A significant increase of distinct growth factors and support of cell proliferation has been shown in ‘platelet releasate supernatant rich in growth factors’ compared to HPL [19]. The current lack of standardized manufacturing protocols for in-house and also commercial HPL products may disable comparability and is still an issue within the research field [20, 21]. Notably, a systematic comparison of three different commercial HPL products for expansion of bone marrow (BM)- and white adipose tissue (WAT)-derived stromal cells revealed sufficient cell proliferation rates and comparable in vitro differentiation capacity, immune phenotypes and genomic stability [22].

The investigation of bioactive molecules promoting in vitro cell expansion demonstrated, that a high fibrinogen concentration in HPL affected the proliferation of WAT- and BM-derived stromal cells [23]. Furthermore, HPL-cultured stromal cells directly bound fibrinogen molecules, leading to altered cytokine expression and immunomodulatory capacities [15]. In a comprehensive review [6] 34% of cited studies used fibrinogen-depleted HPL after addition of calcium chloride [15, 24] or after spontaneous clotting during medium preparation [25]. Fibrinogen-depleted HPL also enables heparin-free cell culture, avoiding putative influences of heparin on differential gene expression of stromal cells [26].

The aim of this study was a systematic comparison of pooled HPL-based medium preparations (pHPL) and two differentially fibrinogen-depleted variants and FBS with respect to the biochemical composition and concentration of growth factors and cytokines. We investigated their specific influence on stromal cell surface marker expression, consumption and secretion of specific growth factors and cytokines during culture, in vitro trilineage differentiation, proliferative and clonogenicity of stromal cells derived from three different tissue sources. In a recently published gene expression analysis, SOX2 and KLF4 were significantly upregulated under pHPL-based culture conditions [26]. These transcription factors, amongst others, are well known to bind specific regulatory elements during mitosis, also referred to mitotic bookmarking, and thus putatively allow an accelerated re-entry to the cell cycle [27, 28]. We therefore examined mRNA expression of SOX2, KLF4, cMYC, OCT4 and Nanog in pHPL- compared to FBS-supplemented culture conditions.

In our study, cell proliferation was significantly enhanced in pHPL-based media independent of fibrinogen and heparin, compared to FBS supplementation. This is in line with previous data and is mainly attributed to abundant platelet-derived growth factors and cytokines [6, 7, 29]. The mRNA expression levels of the mitotic bookmarking factors SOX2, cMYC and KLF4 were significantly elevated in stromal cells cultured in pHPL-based media irrespective of the preparation mode in comparison to FBS-supplemented medium.

## Methods

### Modification of pHPL and medium preparation

Production of pHPL was performed as described previously [10, 11], with some modifications: expired irradiated buffy-coat-pooled platelet concentrates were each derived from 4 healthy blood donors after signed informed consent. The platelet units were lysed on day 5 to 7 by three cycles of freezing/thawing at − 30 °C/37 °C and ten units of mixed ABO blood groups were pooled for one batch of pHPL (consisting of 40 blood donations). After centrifugation (4000×*g*, 15 min at RT) for depletion of platelet fragments, aliquots were stored at − 30 °C until use. Fibrinogen-depleted pHPL ‘serum’ (pHPLS) was prepared by adding 12 mM CaCl_2_ (Merck Millipore, Darmstadt, Germany) to the lysed platelet units after the second freeze/thaw step, incubation for 3 h at 37 °C and overnight at 4 °C. After centrifugation (4000×*g*, 15 min, RT), the fibrin-free supernatant was collected and stored at − 30 °C. Mechanical fibrinogen depletion of pHPL supplemented alpha-modified Minimum Essential Eagle’s Medium (α-MEM, Sigma Aldrich, St. Louis, MO, USA) was performed as previously described [25].

α-MEM with 5.5 mM (N2)-l-Alanyl-l-Glutamin (Dipeptiven^®^, Fresenius Kabi, Graz, Austria) was supplemented (v/v) either with

I. 10% pHPL plus 2 IU/mL heparin (Biochrom, Berlin, Germany),
II. 10% pHPLS,
III. 10% mcpHPL (medium-clotted pHPL after mechanical fibrinogen-depletion [25]), or
IV. 16.5% FBS (Biochrom, Berlin, Germany).

**Fig. 1:**
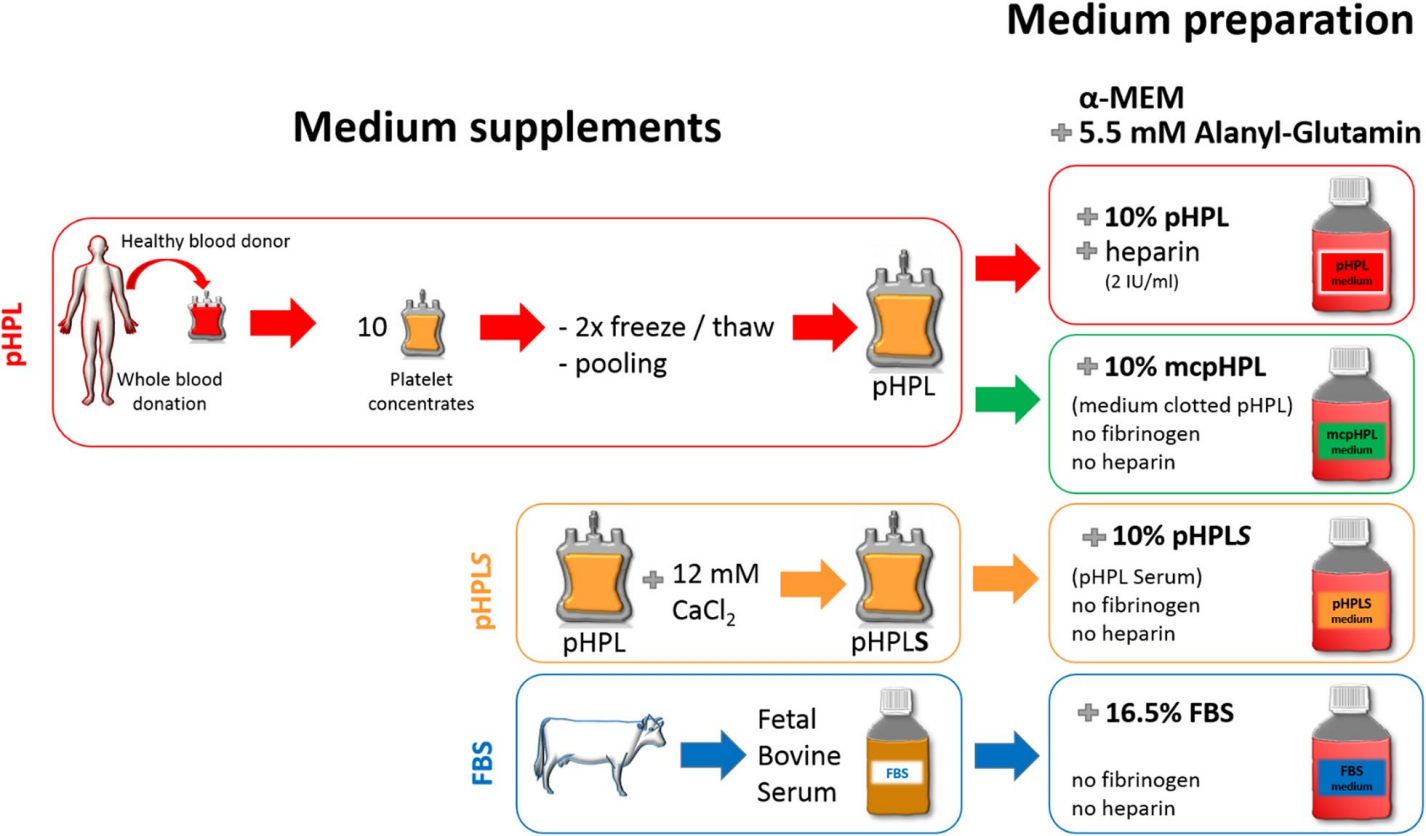
Preparation of different supplements and medium types. Scheme of the production steps of supplements (left) and composition of different pHPL- and FBS-based media (right)

Media were sterile filtered using 0.2 µm filters (Merck Millipore). An overview of the different medium types and their contents and important steps during the production process is given in Fig. 1.

### Biochemical analysis of pHPL preparations, FBS and supplemented media

Total protein, albumin, glucose, total bilirubin, Fe^3+^, Na^+^, K^+^, Cl^−^, Ca^2+^, Mg^2+^, cholesterol and triglycerides of ten batches of pHPL and pHPLS, and six batches of FBS were analyzed automatically (cobas-8000-c702 and ISE Moduls; Roche, Basel, Switzerland). Fibrinogen was measured using the automated hemostasis analyzer BCS XP (Siemens Healthcare, Erlangen, Germany). The Osmomat auto (Gonotec, Berlin, Germany) was employed for analysis of osmolality; pH was determined by RapidPoint RP 500 (Siemens Healthcare).

### Growth factor and cytokine analysis

The concentration of 45 growth factors and cytokines was determined by a multiplex immunoassay (Cytokine/Chemokine/Growth Factor 45-Plex Human Panel 1, Luminex xMAP, Life Science Solutions—Thermo Fisher Scientific, Darmstadt, Germany) according to manufacturer’s instructions. We tested pHPL-, pHPLS-, mcpHPL- and FBS-supplemented medium without cells (‘medium only’) on day 0, after incubation at 37 °C without cells on day 5 and in conditioned medium after culture of BM-, UC- and WAT-derived stromal cells (each 3 donors, measured in duplicates) on day 5. A list of the analyzed parameters is provided in Additional file 1.

### Isolation and culture of stromal cells

Stromal cells were isolated from white adipose tissue (WAT) and umbilical cord (UC) after signed informed consent by the donors as described previously [26, 30, 31]. Bone marrow (BM)-derived stromal cells were purchased from AllCells, (Alameda, CA, USA, http://www.allcells.com/statement-of-ethical-standards/). Cells were isolated and cultured separately in the different medium preparations (for each tissue source n = 3) (I–IV) at 37 °C, 5% CO_2_ and 95% humidity. Antibiotics (100 mg/mL streptomycin and 62.5 mg/mL penicillin, LifeTechnologies, Carlsbad, CA, USA) were used for initial seeding for UC-derived stromal cells only and were removed after 48 h by medium exchange.

### Cell proliferation and colony forming unit (CFU) assays

Stromal cells were cultivated in all medium types for four passages (seeding density 100 cells/cm^2^ in 225 cm^2^ flasks in duplicates). Medium was exchanged on day 2 and day 5. On day 7 cells were trypsinized (TrypLE™ Express, Gibco™ by Life Technologies, Thermo Fisher Scientifics) and total cell counts were determined using C-Chip counting chambers (Bioswisstec, Schaffhausen, Switzerland). Cumulative population doublings were calculated as ln(N)/ln(2) with N being the cell number of detached cells divided by the number of cells seeded. To investigate colony forming capacity, one cell/cm^2^ was seeded in cell culture dishes (diameter 150 mm) and cultured for 14 days. Every 3rd day medium was exchanged. Finally, colonies were fixed in 4% paraformaldehyde (PFA, Sigma Aldrich) and stained with 0.05% Crystal Violet (Sigma Aldrich). Colonies defined with more than 50 cells were counted visually. CFU assays were done in triplicate over four subsequent passages.

### Flow cytometry analysis of cellular surface markers

After passage one in differential culture media, 5 × 10^5^ stromal cells per staining (n = 3 for each tissue source) were re-suspended in 50 µL phosphate buffered saline (PBS, Sigma Aldrich). Cells were mixed with 0.5 µL viability dye (Fixable Viability Dye eFluor™ 520, eBioscience, Thermo Fisher Scientifics) and 10–13 µL antibody mastermix for CD73, CD90, CD105, CD19, CD14, CD34, CD45 and HLA-DR. After incubation (30 min on ice in the dark) and a washing step with PBS, the cell pellet was dissolved in 200 µL PBS. Cells were measured immediately (BD LSRFortessa™, Becton–Dickinson) and results were analyzed with Kaluza Analysis Software (Beckman Coulter, Brea, CA, USA). Details of antibodies and corresponding isotype controls see Additional file 2.

### In vitro differentiation assays

Adipogenic and osteogenic differentiation potential of stromal cells was tested at passage 2. For differentiation, 10^3^ cells/cm^2^ were seeded in 24 well plates in the differential media. After 24 h, medium was replaced by differentiation medium as described [32]. At day 21, cells were fixed using 4% PFA (Sigma Aldrich) and stained with either 0.5% Alizarin Red (Sigma Aldrich) or 1% Sudan III (Sigma Aldrich). Photographs were taken using a PrimoVert Light microscope and an AxioCam ERc5s digital camera (both Zeiss, Oberkochen, Germany). In vitro 3D chondrogenic differentiation of BM-derived stromal cells (n = 3) was performed as previously described [32, 33]. In brief, 5 × 10^5^ cells were seeded onto collagen I-coated (Sigma Aldrich) transwells (Corning, Corning, NY, USA). Cartilage discs were grown in chondrogenic differentiation medium [Dulbecco’s Modified Eagle Medium (DMEM) high glucose (Sigma Aldrich) supplemented with 40 µg/mL l-prolin (Sigma Aldrich), 10^−7^ M dexamethasone (Stem cell technologies, Vancouver, Canada), 25 µg/mL l-ascorbic acid 2-phosphate (Sigma Aldrich), 1x Insulin-transferrin sodium selenite plus linoleic-BSA (ITS + 1) cell culture supplement (Sigma Aldrich), 1x sodium pyruvate (Sigma Aldrich), 1x l-glutamine (Life Technologies), 1x Pen/Strep (Sigma Aldrich) and 10 ng/mL TGF beta (Pepro Tech, London, UK)]. Cultures were incubated at 37 °C with medium being changed every 2nd day. After 3 weeks, the cartilage discs were harvested and weights were measured. Discs were formalin-fixed, paraffin-embedded and processed into 4 μm sections. Deparaffinized and hydrated sections were stained with 0.2% Fast Green (Morphisto, Frankfurt, Germany) and 1.0% Safranin O (Merck) as previously published [26]. Stained sections on slides were scanned automatically in 40× magnification using the Olympus slidescanner VS120 and the Olympus VS-ASW-L100 program (both Olympus, Tokyo, Japan). For quantification of in vitro chondrogenic differentiation, Bern Score evaluation was done by three independent observers as previously published by evaluating the SafraninO/Fast Green stained paraffin sections [34].

### RNA isolation and quantitative real-time PCR (qRT-PCR)

To examine mRNA expression of the transcription factors SOX2, KLF4, cMYC, OCT4 and Nanog, total RNA was isolated from stromal cells (passage 1, n = 3 for each source) cultured in different media using High Pure RNA isolation kit (Roche Diagnostics, Rotkreuz, Switzerland) according to manufacturer’s protocol. cDNA synthesis was done as described previously [35]. qRT-PCR analysis was performed using a LightCycler 480 II and LightCycler 480 SYBR Green I Master reagent (both Roche Diagnostics) according to manufacturer’s instructions. For normalization of sample material, human Glyceraldehyde 3-phosphate dehydrogenase (GAPDH) was used. Data analysis was done as described [36], heat maps were done using ClustVis tool [37]. For qRT-PCR primer sequences are according to Lee et al. [38]. For detailed sequence information see also Additional file 3.

### Statistical analysis

All data are presented as mean ± SD. D’Agostino and Pearson omnibus normality test was used to determine if data followed a Gaussian distribution. Data were analyzed using one-way ANOVA, two-way ANOVA and Tukey’s test or unpaired t-test, with GraphPad Prism 7 (GraphPad Software, La Jolla, CA, USA) with p < 0.05 being considered as significant.

## Results

### Biochemical composition of pHPL, pHPLS, FBS and supplemented media

In Table 1 the concentration of biochemical parameters of pHPL and pHPLS (10 lots), FBS (6 lots) and reference human blood values are shown. Comparing pHPL and pHPLS, fibrinogen was significantly reduced (p < 0.001), whereas osmolality, Cl^−^, Ca^2+^ and Mg^2+^ were significantly increased in pHPLS (p < 0.001). Compared to standard blood values, glucose and Na^+^ were increased in pHPL and pHPLS. All pHPL preparations showed significantly increased levels for total protein, albumin, glucose, cholesterol and Na^+^ (p < 0.001) compared to FBS, while K^+^ and Fe^3+^ were significantly reduced (p < 0.001). Furthermore, biochemical parameters tested for basal αMEM and αMEM supplemented with pHPL, pHPLS, mcpHPL and FBS are shown. Total protein, glucose, Fe^3+^, Ca^2+^ and K^+^ were significantly different in all pHPL medium preparations compared to FBS-medium (p-values as indicated).

**Table 1:** Comparison of biochemical parameter concentrations tested in pHPL, pHPLS and FBS, standard human blood values (pHPL vs. pHPLS: ^###^p < 0.001; pHPL or pHPLS vs. FBS: *p < 0.05, ***p < 0.001) as well as basic α-MEM and α-MEM supplemented with 10% pHPL, pHPLs and mcpHPL or 16.5% FBS (pHPL-media vs. FBS-medium: ^+^p < 0.05, ^++^p < 0.01 and ^+++^p < 0.001)

	Supplements			Standard blood values	Medium	Medium supplemented with			
	pHPL	pHPLS	FBS		α-MEM	pHPL (10%)	pHPLS (10%)	mcpHPL (10%)	FBS (16.5%)
pH	7.4 ± 0.1***	7.4 ± 0.1***	7.7 ± 0.1	7.4–7.5	7.6	7.8 ± 0.0	7.8 ± 0.0	7.8 ± 0.0	7.6 ± 0.0
Osmolality (mosmol/kg)	313 ± 4^###,^*	337 ± 6***	307 ± 0	280–300	293	290 ± 8^+^	297 ± 4	294 ± 2	299 ± 6
Total Protein [g/dL]	6.2 ± 0.2***	6.0 ± 0.2***	3.8 ± 0.1	6.5–8.5	n.d.	0.7 ± 0.1^+++^	0.7 ± 0.0^+++^	0.7 ± 0.1^+++^	0.3 ± 0.1
Albumin [g/dL]	4.0 ± 0.2***	4.0 ± 0.2***	2.4 ± 0.1	3.5–5.5	n.d.	0.2 ± 0.2	0.3 ± 0.1	0.3 ± 0.1	0.3 ± 0.1
Glucose [mg/dL]	298 ± 9***	290 ± 10***	129 ± 8	70–100	103	113 ± 3^++^	115 ± 2^++^	114 ± 3^++^	103 ± 4
Fibrinogen [mg/dL]	234 ± 58^###,^***	< 30	< 30	200–400	n.d.	n.d.	n.d.	n.d.	n.d.
Triglyceride [mg/dL]	80 ± 10	78 ± 7	75 ± 2	75–200	n.d.	7.4 ± 0.4	8.5 ± 0.5	9.3 ± 2.5	14 ± 6.0
Cholesterol [mg/dL]	161 ± 11***	159 ± 6***	37 ± 2	120–250	10.0	17 ± 4	19 ± 4	17 ± 4	9 ± 3.0
Na^+^ [mmol/L]	172 ± 2***	171 ± 2***	138 ± 2	135–148	148	142 ± 6	145 ± 3	143 ± 3	143 ± 0
K^+^ [mmol/L]	4.6 ± 0.1***	4.6 ± 0.1***	11.9 ± 0.4	3.6–5.0	5.5	5.2 ± 0.2^+++^	5.3 ± 0.1^+++^	5.2 ± 0.1^+++^	6.3 ± 0.3
Cl^−^ [mmol/L]	71 ± 1^###,^***	93 ± 3	93 ± 1	97–108	126	113 ± 5	118 ± 3	115 ± 2	119 ± 1
Ca^2+^ [mmol/L]	2.1 ± 0.1^###,^*	13.8 ± 1.7***	3.7 ± 0.1	2.1–2.6	2.0	1.8 ± 0.1^+^	2.9 ± 0.1^+++^	1.8 ± 0.1^+^	2.1 ± 0.1
Mg^2+^ [mmol/L]	0.8 ± 0.0^###,^***	1.3 ± 0.1	1.3 ± 0.0	0.8–1.0	1.0	0.8 ± 0.0	0.8 ± 0.0	0.8 ± 0.0	0.9 ± 0.0
Fe^3+^ [mmol/L]	78 ± 9***	76 ± 8***	184 ± 2	60–150	n.d.	8.0 ± 0.0^++^	7.7 ± 1.2^++^	8.0 ± 1.0^++^	25 ± 8

Data are shown as mean ± SD

*n.d.* not detected

### Growth factor and cytokine analysis

The concentration of 45 cytokines and growth factors was analyzed in differentially prepared 10% pHPL- and 16.5% FBS-supplemented ‘medium only’ (each 1 batch) on day 0 and day 5, and in the corresponding conditioned medium after 5 days of culturing BM-, WAT- and UC-derived stromal cells (3 donors each) to enable discrimination between secreted and consumed factors. A complete list of the results of cytokine and growth factor analysis is shown in Additional file 4. Notably, none of the proteins was detected in FBS-supplemented ‘medium only’ on day 0 and day 5, due to the fact that the multiplex assay is not specific for bovine proteins.

**Fig. 2:**
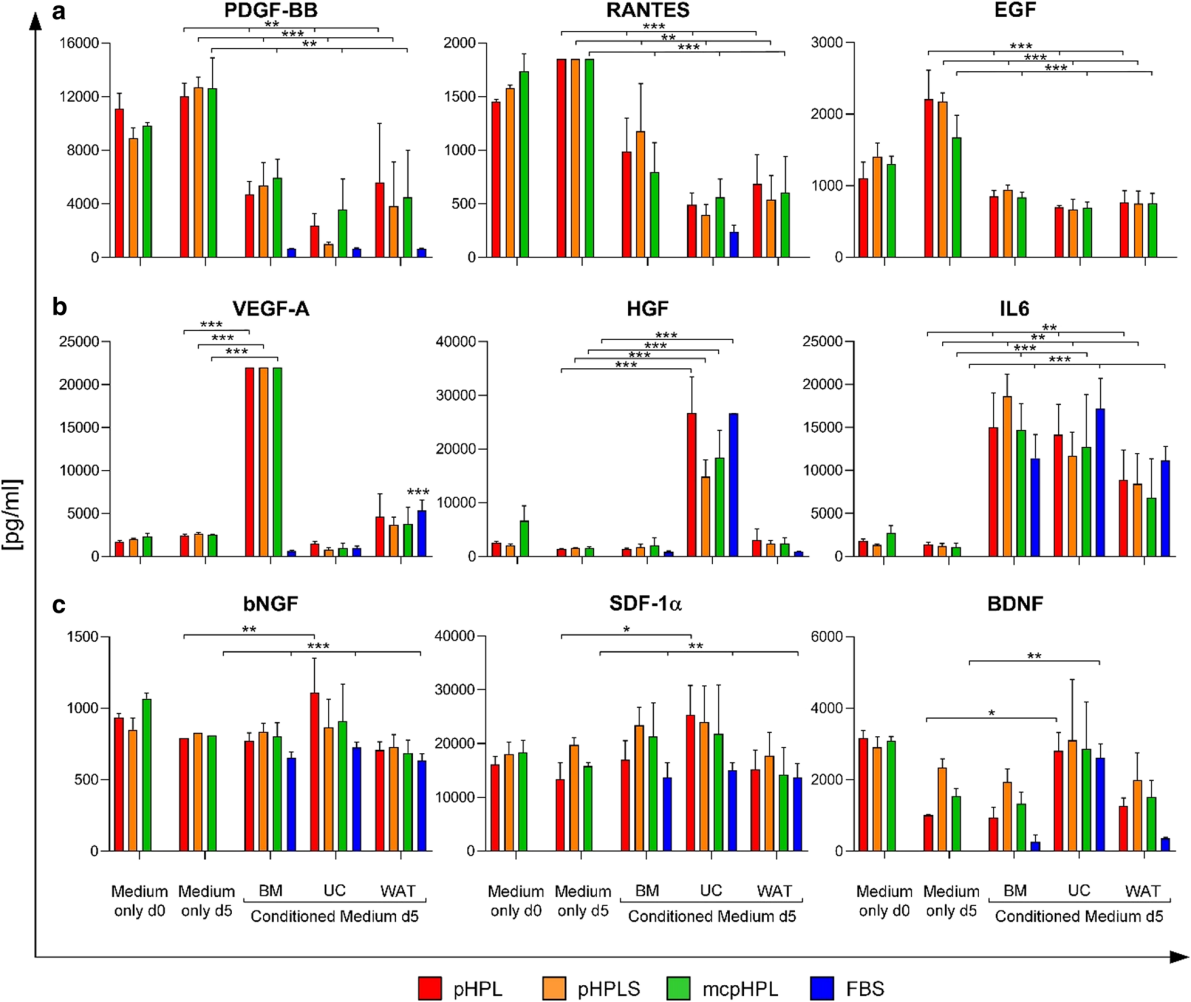
Comparison of growth factor and cytokine content in supplemented ‘medium only’ day 0 and day 5 and conditioned medium day 5. Concentration of PDGF-BB, RANTES, EGF, VEGF-A, HGF, IL6, bNGF, SDF-1α and BDNF in differentially supplemented ‘medium only’ day 0 and day 5, and conditioned medium day 5 after culturing BM-, UC- and WAT-derived stromal cells, analyzed by multiplex analysis. Data are shown as mean ± SD of three cell donors each, measured in duplicates. Two-way ANOVA was used to determine statistically significant differences (*p < 0.05, **p < 0.01, ***p < 0.001 compared to ‘medium only’ day 5)

As shown in Fig. 2 and Additional file 4, fibrinogen depletion of pHPL had no statistically significant influence on the concentration of analyzed growth factors and cytokines (‘medium only day’ 0). In Fig. 2 a subset of the analyzed growth factors and cytokines is depicted as examples. Compared to ‘medium only’ day 5, in conditioned medium PDGF-BB, RANTES and EGF were consumed by stromal cells (Fig. 2a). In contrast, VEGF-A, HGF and IL6 were significantly enhanced after 5 days compared to ‘medium only’, indicating that these factors were produced by stromal cells in a source-dependent manner (Fig. 2b). High amounts of VEGF-A were produced by BM-but not by UC- and WAT-derived stromal cells, whereas HGF was produced by UC-derived stromal cells only. Elevated levels of IL6 were detected in all conditioned media, irrespective of the cell origin. The levels of bNGF, SDF-1α and BDNF were maintained in pHPL-based media and increased in FBS-medium putatively due to simultaneous secretion and consumption (Fig. 2c).

### Stromal cell proliferation and cloning efficiency

**Fig. 3:**
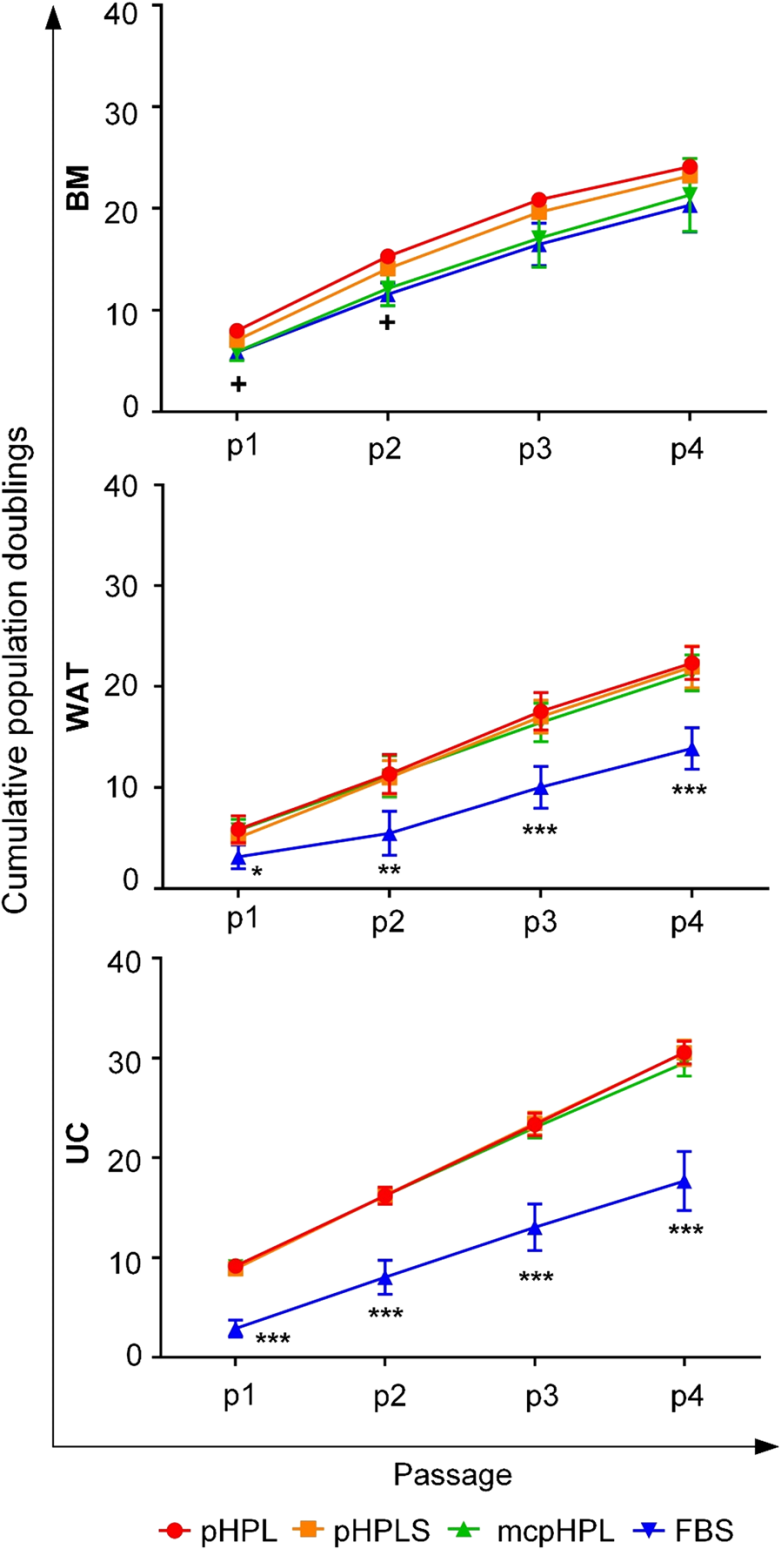
Proliferation capacity is enhanced for different stromal cells in pHPL-based media compared to FBS-supplemented medium. Proliferative capacity of stromal cells expanded in either pHPL- (red), pHPLS- (orange) or mcpHPL- (green) compared to FBS- (blue) medium of UC- and WAT-derived stromal cells. Data are shown as cumulative population doublings (cPD) of three independent stromal cell donations for each tissue source tested in duplicates ± SD (^+^p < 0.05 comparing pHPL/pHPLS to FBS-supplementation; *p < 0.05, **p < 0.01, ***p < 0.001, all pHPL-media compared to FBS-supplementation)

Cumulative population doublings (cPD) of BM-, WAT- and UC-derived stromal cells cultured in the three different pHPL-media were similar over four passages within 28 days of culture but as expected, significantly increased compared to culture in FBS-medium (Fig. 3). UC- and WAT-derived stromal cells revealed significantly enhanced proliferation rates in every pHPL-based medium compared to FBS in all passages (p < 0.05, p < 0.01 and p < 0.001). In contrast, BM-derived stromal cells revealed significantly higher proliferation (p < 0.05) only in pHPL- and pHPLS-but not in mcpHPL-based medium compared to FBS-supplemented medium at early passages 1 and 2.

We did not detect a significantly different clonogenicity between fibrinogen-containing and fibrinogen-depleted pHPL-based media in BM- and WAT-derived stromal cells. Clonogenicity of UC-derived stromal cells was significantly elevated in fibrinogen-depleted media (pHPLS- and mcpHPL) compared to standard pHPL-medium (p < 0.01), but only in passage 1. Furthermore, clonogenicity of BM-derived stromal cells was significantly decreased in pHPL-based media compared to FBS. In contrast, we observed a significantly higher colony forming capacity of UC-derived stromal cells expanded in pHPL-media compared to FBS-based cultivation (p < 0.05). Cloning efficiency of pHPL- and FBS-cultivated WAT-stromal cells was comparable in early passages 1 and 2, but significantly decreased (p < 0.05) in pHPL- compared to FBS-medium at later passages 3 and 4 (Additional file 5A and B).

### Immunophenotype and in vitro differentiation of BM-, WAT- and UC-derived stromal cells

In order to investigate whether the different preparation techniques of pHPL-based media impact stromal cells, we first performed flow cytometry analysis of stromal cells cultivated in pHPL-, pHPLS-, mcpHPL- or FBS-media. Our data revealed that the canonical surface expression pattern of CD73^+^/90^+^/105^+^ and CD14^−^/19^−^/34^−^/45^−^/HLA-DR^−^ (Additional file 6A) is maintained irrespective of the pHPL-medium type. No statistically significant differences in surface marker expression of stromal cells cultured under different conditions were observed. Independent of culture media, also the in vitro osteogenic and adipogenic differentiation potential was maintained (Additional file 6B). As previous studies have revealed that WAT- and UC-derived stromal cells do not differentiate in vivo into the chondrogenic lineage [2, 32, 39], chondrogenic differentiation capacity was analyzed for BM-derived stromal cells only. Chondrogenesis was confirmed by SafraninO/FastGreen staining (Additional file 7A). For quantification, the weights of the 3D cartilage discs were measured (Additional file 7B) and for an independent evaluation the Bern scoring [34], a visual histological grading system, was applied (Additional file 7C). Even though FBS-cultivated cartilage discs showed significantly lower weights compared to discs in pHPLS- and mcpHPL-culture (p < 0.05 and p < 0.001, respectively), the Bern Scoring revealed no significant differences between the culture conditions. In summary, in vitro tri-lineage differentiation of stromal cells was maintained in all tested culture conditions.

### Analysis of NANOG, SOX2, KLF4, cMYC and OCT4 mRNA expression

**Fig. 4:**
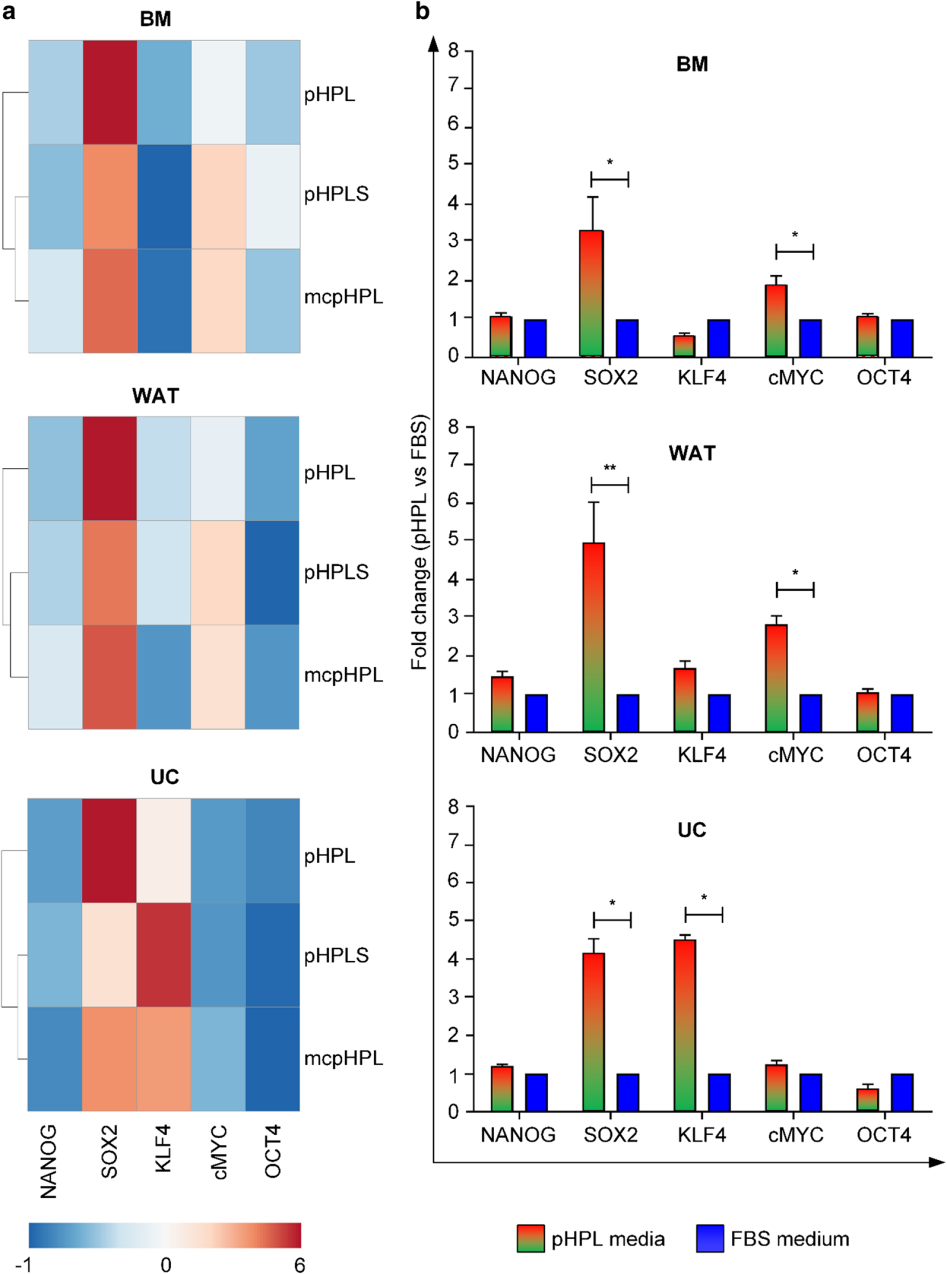
Upregulation of mRNA expression of distinct transcription factors in pHPL-media. **a** Heat map depicting the fold change mRNA expression of NANOG, SOX2, KLF4, cMYC and OCT4 in stromal cells cultivated in pHPL-, pHPLS- and mcpHPL-based medium with red color indicating up- and blue color downregulation compared to FBS medium. **b** mRNA expression of selected transcription and mitotic bookmarking factors in BM-, WAT- and UC-derived stromal cells cultured in pHPL-based media (summarized as red/orange/green bar) compared to FBS (blue bar). Data shown are mean fold change values of three individual donors for each tissue source measured in duplicates (*p < 0.05, **p < 0.01)

Comparing pHPL- with FBS-supplemented culture conditions, the cellular mRNA expression of several mitotic bookmarking factors was significantly enhanced (p < 0.05). Our analysis revealed, that the mRNA levels of SOX2 were significantly augmented in stromal cells irrespective of the tissue source and pHPL-medium preparation mode (Fig. 4a). Like SOX2, there are several other transcription factors acting as mitotic bookmarking factors such as cMYC, KLF4, NANOG, GATA1, HSF2, FOXA1, OCT4, RUNX2 and TLE1 (for review see Festuccia et al. [27]). Since the expression of SOX2, KLF4, NANOG, OCT4 and cMYC is also described to be characteristic for pluripotent progenitor cells [40–42], we investigated the mRNA expression levels of this selected subset of factors. The expression of cMYC was significantly enhanced (p < 0.05) in BM- and WAT-, but not in UC-derived stromal cells (Fig. 4a, b). In contrast, the transcription factor KLF4, was enhanced in UC-derived stromal cells only (Fig. 4a, b, p < 0.05). Our data further revealed that OCT4 and NANOG expression were unaffected by different culture conditions.

## Discussion

Manufacturing protocols for stromal cell therapeutics are highly variable. Albeit FBS-driven stromal cell culture is still common practice for many clinical trials, there are international efforts ongoing to reach standardized production and definition of quality parameters for HPL [21, 43].

In this study, we tested the concentration of several human blood associated biochemical parameters, growth factors and cytokines in pHPL, pHPLS and FBS as well as the corresponding supplemented media including mechanically fibrinogen-depleted pHPL-based medium. Comparing pHPL and pHPLS, significant differences were detected for osmolality, fibrinogen, Ca^2+^, Cl^−^ and Mg^2+^, which can be ascribed to the different preparation steps for pHPLS. As expected, most of the biochemical parameters of pHPL and pHPLS were comparable to human reference blood values. Glucose and Na^+^ levels were increased according to citrate phosphate dextrose (CPD) solution as anticoagulant in blood collection bags. Similar results for biochemical analysis of pHPL/pHPLS were observed by Shanskii et al. [44] and Pierce et al. [45]. However, these studies lack a direct comparison of different pHPL preparation modes. Our data reveal, that the mode of pHPL preparation has no relevant influence on biochemical properties of HPL-supplemented medium, since the majority of parameters tested was not altered or at least balanced by dilution. Higher Fe^3+^ levels in FBS compared to pHPL go ahead with the observation that bovine fetuses show elevated Fe^3+^ stores [46]. In addition, high levels of K^+^ were detected in FBS, most likely due to cell lysis caused by the crude mode of collection.

Previous proteomic analyses of different platelet derivatives showed that platelet-derived factors may differentially influence proliferation of stromal cells. These include cytokines and growth factors such as FGF, PDGFs, TGF-beta, GM-CSF, RANTES, IGF, HGF and different interleukins [13, 15, 23, 24]. Three independent studies comparing various platelet products showed similar as well as dissimilar levels of different cytokines and growth factors [13, 15, 45]. In this study, we found various concentrations of analyzed cytokines and growth factors but no statistically significant difference comparing pHPL-based media before and after fibrinogen depletion. These divergent observations further highlight the need for an extended standardization of pHPL as raw material [21, 43].

The therapeutic effects of stromal cells are supposed to be caused by direct cell interaction but also paracrine signaling. The cytokine and growth factor milieu during cultivation may support stromal cell immune-modulatory capacity, facilitate efficient engraftment and support wound healing [47–49]. We therefore investigated whether different pHPL- and FBS-based culture conditions affect the secretion of bio-active molecules by BM-, UC- and WAT-derived stromal cells. Our data revealed three different groups of cytokines and growth factors: Factors that are pHPL-borne and consumed by stromal cells (e.g., PDGF-BB, RANTES and EGF), factors that were mainly secreted by stromal cells (e.g., VEGF-A, HGF and IL6) and factors that were putatively consumed and secreted (e.g., bNGF, SDF-1α and BDNF). The concentration of these factors was not significantly different in pHPL-supplemented conditioned media from all tested cell types.

Stromal cell proliferation was still significantly enhanced in all pHPL- compared to FBS-media, due to abundant growth factors. No statistically significant differences were found between different pHPL-based medium types for the colony forming capacity of stromal cells. However, in comparison to FBS, tissue source dependent effects were observed: While clonogenicity decreased in BM-stromal cells, it was enhanced in UC-derived stromal cells. WAT-derived stromal cells revealed comparable numbers of colonies at early passages 1 and 2 and revealed decreased numbers in pHPL-based media at later passages 3 and 4. Despite cell source dependent effects, the colony forming capacity of stromal cells was well maintained in pHPL-based media, irrespective of fibrinogen and heparin. Furthermore, the characteristic surface marker expression pattern as well as the in vitro osteogenic, adipogenic and chondrogenic differentiation potential of stromal cells were not influenced by different pHPL-based media, as also shown previously [26].

As reviewed recently [28], rapid proliferation of stem/progenitor cells is tightly linked to their pluripotent state. During development, the differentiation and increasing specialization of stem/progenitor cells is accompanied by prolonged cell cycle phases, resulting in reduced proliferation rates. These changes in the cell cycle are still poorly understood. Especially the role of so called pluripotency factors, often also associated with modified cell cycle checkpoints in stem/progenitor cells as well as malignant cells, remains elusive [28]. In this study we investigated the impact of culture conditions on the expression of mitotic bookmarking factors SOX2, KLF4, cMYC, OCT4 and NANOG in stromal cells from different sources, as several studies have shown that PDGF-BB, which is a central component of HPL [6], directly influences the expression of KLF4: The KLF4 promoter has three Sp1 binding sites, which are required for both baseline and PDGF-BB-induced KLF4 promoter activity in murine smooth muscle cells [50]. Deaton et al. showed that the knock-down of the activating transcription factor Sp1 prevented efficiently the PDGF-BB induced increase of endogenous KLF4 expression [51]. Furthermore, PDGF-BB directly increased KLF4 promoter activity and thereby enhanced the expression of KLF4 in human primary pulmonary artery endothelial cells [52]. Liu et al. demonstrated the ability of KLF4 and other factors to remain associated with chromatin during cell division at a single cell level of mouse embryonic stem cells, thus suggesting a potential bookmarking function [53]. Furthermore, KLF4 is thought to play a role in the control of G1-S-transition of the cell cycle [54, 55].

Sacca et al. identified PDGF-responsive elements in the cMYC promoter already [56]. PDGF enhanced the expression of cMYC and stimulated the cMYC promoter in a Src-dependent manner [57, 58]. During mitosis, also cMYC, known as key regulator of cellular proliferation [59, 60], was shown to bind actively to chromatin sites associated with genes being important for cell cycle [61].

In addition to PDGF, also other HPL-borne factors such as LIF [62] and EGF [63] were shown to activate KLF4 and also SOX2. In our study pHPL-supplemented media significantly enhanced mRNA expression of SOX2, cMYC and KLF4. These factors are known as mitotic bookmarking factors [53, 61, 64], associating with chromatin during mitosis in a highly dynamic manner and accelerating gene reactivation after mitosis during early G1 phase [27, 28]. This bookmarking may be important for maintaining a global accessibility to chromatin, thereby allowing a quick reassembly of regulatory complexes at promoters, fostering a fast reactivation of transcription [27]. Our data revealed that SOX2 was significantly enhanced in all stromal cell types. SOX2 is known to be involved in embryonic development, the determination of cell fate and also to bookmark mitotic chromatin of pluripotent and non-pluripotent cells in order to facilitate a rapid re-establishment of gene expression after mitosis, allowing to enter a new cell cycle more quickly [27, 28, 64, 65]. Our finding is in line with previous data, showing that SOX2 expression is enhanced in human dental pulp stem cells cultured in HPL [66]. The absence of SOX2 at the M-G1 transition was shown to result in a decreased capacity of self-renewal of murine embryonic stem cells [65]. Furthermore, the expression levels of SOX2, OCT4 and NANOG were directly correlated with the cell cycle velocity, with high expression corresponding to strong self-renewing capacities in murine embryonic cells [67]. The exact mechanisms are still not fully understood, but together with its interaction partner OCT4, SOX2 regulates CyclinD/Cdk activity, ensuring a shorter G1 phase and therefore contributing to higher proliferation rates [68]. In our study we also found cMYC mRNA expression to be significantly enhanced in BM- and WAT- but not in UC-derived stromal cells cultured in pHPL compared to FBS. These results are in line with previous data showing that WAT-stromal cells expressed SOX2, NANOG, KLF4, cMYC and OCT4 when cultured in pHPL [69]. In contrast, we found KLF4 to be significantly enhanced only in UC-derived stromal cells cultured in pHPL compared to FBS culture, going in line with previous reports about tissue-dependent differences of stromal cells [2, 3, 32, 39]. In summary, HPL-borne growth factors such as PDGF-BB and LIF may stimulate the expression of “solid candidates” [27] for mitotic bookmarking, thereby allocating a growth promoting stimulus for stromal cells.

## Conclusions

Clonogenicity and in vitro differentiation of stromal cells was well maintained in all pHPL-supplemented media, independent of fibrinogen. Compared to FBS, all pHPL-preparations significantly enhanced cell proliferation corresponding to enhanced mRNA expression of the transcription and mitotic bookmarking factors SOX2, cMYC and KLF4. Our data strongly indicate for the first time, that this lead role of pHPL might be explained by the enhanced expression of mitotic bookmarking factors enabling an accelerated re-entry to the cell cycle. The significance of our observation for in vivo function of stromal cell therapeutics has to be further investigated.

## Supplementary information

**Additional file 1.** List of analyzed cytokines, chemokines and growth factors in alphabetical order included in the Cytokine/Chemokine/Growth Factor 45-Plex Human Panel 1 (EPX450-12171-901).

**Additional file 2.** List of specific antibodies and isotype controls used for flow cytometry analysis.

**Additional file 3.** Primer sequences used for quantitative RT-PCR according to Lee et al. 2010 [38].

**Additional file 4.** Complete list of cytokine and growth factor concentration (pg/mL) analyzed in differentially supplemented ‘medium only’ day 0 and day 5, and corresponding conditioned medium after 5 days.

**Additional file 5.** Cloning efficiency of stromal cells in different pHPL-media reveals tissue-source dependent effects.

**Additional file 6.** Immunophenotype and in vitro osteogenic and adipogenic differentiation of stromal cells.

**Additional file 7.** In vitro chondrogenic differentiation of bone marrow-derived stromal cells.

## Data Availability

All data generated or analyzed in this study are published in this article (and its additional information files).

## Abbreviations

BM: bone marrow
CFU: colony forming unit
cMYC: MYC proto-oncogene, BHLH transcription factor
FBS: fetal bovine serum
FOXA1: forkhead box protein A1
GATA1: GATA binding protein 1
HSF2: heat shock transcription factor 2
KLF4: Kruppel like factor 4
mcpHPL: medium clotted pHPL
OCT4: octamer-binding protein 4, POU domain, class 5, transcription factor 1
pHPL: pooled human platelet lysate
pHPLS: pHPL serum
RUNX2: RUNX family transcription factor 2
SOX2: SRY (sex determining region Y)-box 2
SP1: specificity protein 1
TLE1: transducin like enhancer of split 1
UC: umbilical cord
WAT: white adipose tissue
