# Supplementary material for: Upregulation of mitotic bookmarking factors during enhanced proliferation of human stromal cells in human platelet lysate
Source: J Transl Med. 2019 Dec 30;17:432. doi: 10.1186/s12967-019-02183-0 (PMC6936143; doi:10.1186/s12967-019-02183-0)
Supplement: Supplementary file 1 — Additional file 1. List of analyzed cytokines, chemokines and growth factors in alphabetical order included in the Cytokine/Chemokine/Growth Factor 45-Plex Human Panel 1 (EPX450-12171-901). [file 12967_2019_2183_MOESM1_ESM.docx]

| **Parameter** | |
| --- | --- |
| **BDNF** | Brain Derived Neurotrophic Factor |
| **bNGF** | Beta Nerve Growth Factor |
| **EGF** | Epidermal Growth Factor |
| **CCL11, Eotaxin** | C-C Motif Chemokine Ligand 11 |
| **FGF-2** | Fibroblast Growth Factor 2 |
| **GM-CSF** | Granulocyte-Macrophage Colony-Stimulating Factor |
| **CXCL1, GRO α** | C-X-C Motif Chemokine Ligand 1, Growth-Regulated Oncogene-alpha |
| **HGF** | Hepatocyte Growth Factor |
| **IFN α** | Interferon alpha 1 |
| **IFNG** | Interferon gamma |
| **IL-10** | Interleukin 10 |
| **IL-12 p70** | Interleukin 12 p70 |
| **IL-13** | Interleukin 13 |
| **IL-15** | Interleukin 15 |
| **IL-17A** | Interleukin 17A |
| **IL-18** | Interleukin 18 |
| **IL-1 α** | Interleukin 1 alpha |
| **IL-1 β** | Interleukin 1 beta |
| **IL-1RA** | Interleukin 1 Receptor Type 1 |
| **IL-2** | Interleukin 2 |
| **IL-21** | Interleukin 21 |
| **IL-22** | Interleukin 22 |
| **IL-23** | Interleukin 23 |
| **IL-27** | Interleukin 27 |
| **IL-31** | Interleukin 31 |
| **IL-4** | Interleukin 4 |
| **IL-5** | Interleukin 5 |
| **IL-6** | Interleukin 6 |
| **IL-7** | Interleukin 7 |
| **IL-8** | Interleukin 8 |
| **IL-9** | Interleukin 9 |
| **CXCL10, IP-10** | C-X-C Motif Chemokine Ligand 10, Interferon gamma-Induced Protein 10 |
| **LIF** | Leukemia Inhibitory Factor |
| **CCL2, MCP-1** | C-C Motif Chemokine Ligand 2, Monocyte Chemotactic Protein 1 |
| **CCL3, MIP-1 α** | C-C Motif Chemokine Ligand 3, Macrophage Inflammatory Protein 1-Alpha |
| **CCL4, MIP-1 β** | C-C Motif Chemokine Ligand 4, Macrophage Inflammatory Protein 1-Beta |
| **PDGF-BB** | Platelet Derived Growth Factor BB |
| **PIGF-1** | Placental Growth Factor |
| **CCL5, RANTES** | C-C Motif Chemokine Ligand 5, Regulated on Activation, Normal T Cell Expressed and Secreted |
| **KITLG, SCF** | KIT-Ligand, Stem Cell Factor |
| **CXCL12, SDF-1α** | C-X-C Motif Chemokine Ligand 12, Stromal cell-derived factor 1alpha |
| **TNF α** | Tumor Necrosis Factor-Alpha |
| **LTA, TNF β** | Lymphotoxin Alpha, Tumor Necrosis Factor-Beta |
| **VEGF-A** | Vascular Endothelial Growth Factor A |
| **VEGF-D** | Vascular Endothelial Growth Factor D |

**Additional File 1**: List of analyzed cytokines, chemokines and growth factors in alphabetical order included in the Cytokine/Chemokine/Growth Factor 45-Plex Human Panel 1 (EPX450-12171-901), Life Science Solutions – Thermo Fisher Scientific.
