# Supplementary material for: Upregulation of mitotic bookmarking factors during enhanced proliferation of human stromal cells in human platelet lysate
Source: J Transl Med. 2019 Dec 30;17:432. doi: 10.1186/s12967-019-02183-0 (PMC6936143; doi:10.1186/s12967-019-02183-0)
Supplement: Supplementary file 2 — Additional file 2. List of specific antibodies and isotype controls used for flow cytometry analysis. [file 12967_2019_2183_MOESM2_ESM.docx]

| **Antibody** | **Dye** | **Type** | **Clone** | **Company** | **µg/mL** |
| --- | --- | --- | --- | --- | --- |
| **CD90** | BUV395 | anti human, mouse IgG1, kappa | 5E10 | BD (#563804) | 200 |
| **Isotype Control** | BUV395 | mouse IgG1, kappa | X40 | BD (#563547) | 200 |
| **CD73** | PE | anti human, mouse IgG1, kappa | AD2 | BD (#550257) | 12.5 |
| **Isotype Control** | PE | mouse IgG1 kappa | MOPC-21 | BD (#555749) | 12.5 |
| **CD105** | eF450 | anti human, mouse IgG1, kappa | SN6 | eBio (#48-1057) | 50 |
| **Isotype Control** | eF450 | mouse IgG1 kappa | P3.6.2.8.1 | eBio (#48-4714) | 50 |
| **CD19** | BUV395 | anti human, mouse IgG1, kappa | SJ25C1 | BD (#563549) | 200 |
| **Isotype Control** | BUV395 | mouse IgG1, kappa | X40 | BD (#563547) | 200 |
| **CD14** | PE | anti human, mouse IgG2b, kappa | MφP9 | BD (#347497) | 50 |
| **Isotype Control** | PE | mouse IgG2b, kappa | MPC-11 | BD (#559529) | 50 |
| **CD34** | PE-Cy7 | anti human, mouse IgG1, kappa | 8G12 | BD (#348791) | 50 |
| **Isotype Control** | PE-Cy7 | mouse IgG1, kappa | MOPC-21 | BD (#557872) | 50 |
| **CD45** | APC | anti human, mouse IgG1 kappa | HI30 | BD (#555485) | 3 |
| **Isotype Control** | APC | mouse IgG1 kappa | MOPC-21 | BD (#555751) | 3 |
| **HLA-DR** | eF450 | anti human, mouse IgG2a kappa | L243 | eBio (#48-9952) | 25 |
| **Isotype Control** | eF450 | mouse IgG2a kappa | eBM2a | eBio (#48-4724) | 25 |

**Additional File 2**: List of specific antibodies and isotype controls used for flow cytometry analysis. BD (Becton Dickinson); eBio (eBioscience, San Diego, CA).
