# Supplementary material for: Upregulation of mitotic bookmarking factors during enhanced proliferation of human stromal cells in human platelet lysate
Source: J Transl Med. 2019 Dec 30;17:432. doi: 10.1186/s12967-019-02183-0 (PMC6936143; doi:10.1186/s12967-019-02183-0)
Supplement: Supplementary file 3 — Additional file 3. Primer sequences used for quantitative RT-PCR according to Lee et al. 2010 [38]. [file 12967_2019_2183_MOESM3_ESM.docx]

| Gene | Primer | Sequence (5‘-3‘) |
| --- | --- | --- |
| **NANOG** | forward | TTTGGAAGCTGCTGGGGAAG |
| Homeobox Transcription Factor Nanog | reverse | GATGGGAGGAGGGGAGAGGA |
| **SOX2** | forward | GGGAAATGGGAGGGGTGCAAAAGAGG |
| SRY (Sex Determining Region Y)-Box 2 | reverse | TTGCGTGAGTGTGGATGGGATTGGTG |
| **KLF4** | forward | TCTCCAATTCGCTGACCCATCCTCC |
| Kruppel Like Factor 4 | reverse | AGGGAGCCGTCGGAGGGGGAGCG |
| **cMYC** | forward | GGACGACGAGACCTTCATCAA |
| MYC Proto-Oncogene, BHLH Transcription Factor | reverse | CCAGCTTCTCTGAGACGAGCTT |
| **OCT4/POU5F1** | forward | GACAGGGGGAGGGGAGGAGCTAGG |
| POU Domain, Class 5, Transcription Factor 1 | reverse | CTTCCCTCCAACCAGTTGCCCCAAAC |

**Additional File 3**: Primer sequences used for quantitative RT-PCR according to Lee et al., 2010 [38].
