# Supplementary material for: Upregulation of mitotic bookmarking factors during enhanced proliferation of human stromal cells in human platelet lysate
Source: J Transl Med. 2019 Dec 30;17:432. doi: 10.1186/s12967-019-02183-0 (PMC6936143; doi:10.1186/s12967-019-02183-0)
Supplement: Supplementary file 4 — Additional file 4. Complete list of cytokine and growth factor concentration (pg/mL) analyzed in differentially supplemented ‘medium only’ day 0 and day 5, and corresponding conditioned medium after 5 days. [file 12967_2019_2183_MOESM4_ESM.docx]

**Additional File 4**: Complete list of cytokine and growth factor concentration (pg/mL) analyzed in differentially supplemented ‘medium only’ day 0 and day 5, and corresponding conditioned medium after 5 days of culturing BM-, WAT- and UC-derived stromal cells (n.d., not detected; n.a., not assessable).
