# Supplementary material for: Upregulation of mitotic bookmarking factors during enhanced proliferation of human stromal cells in human platelet lysate
Source: J Transl Med. 2019 Dec 30;17:432. doi: 10.1186/s12967-019-02183-0 (PMC6936143; doi:10.1186/s12967-019-02183-0)
Supplement: Supplementary file 5 — Additional file 5. Cloning efficiency of stromal cells in different pHPL-media reveals tissue-source dependent effects. [file 12967_2019_2183_MOESM5_ESM.docx]

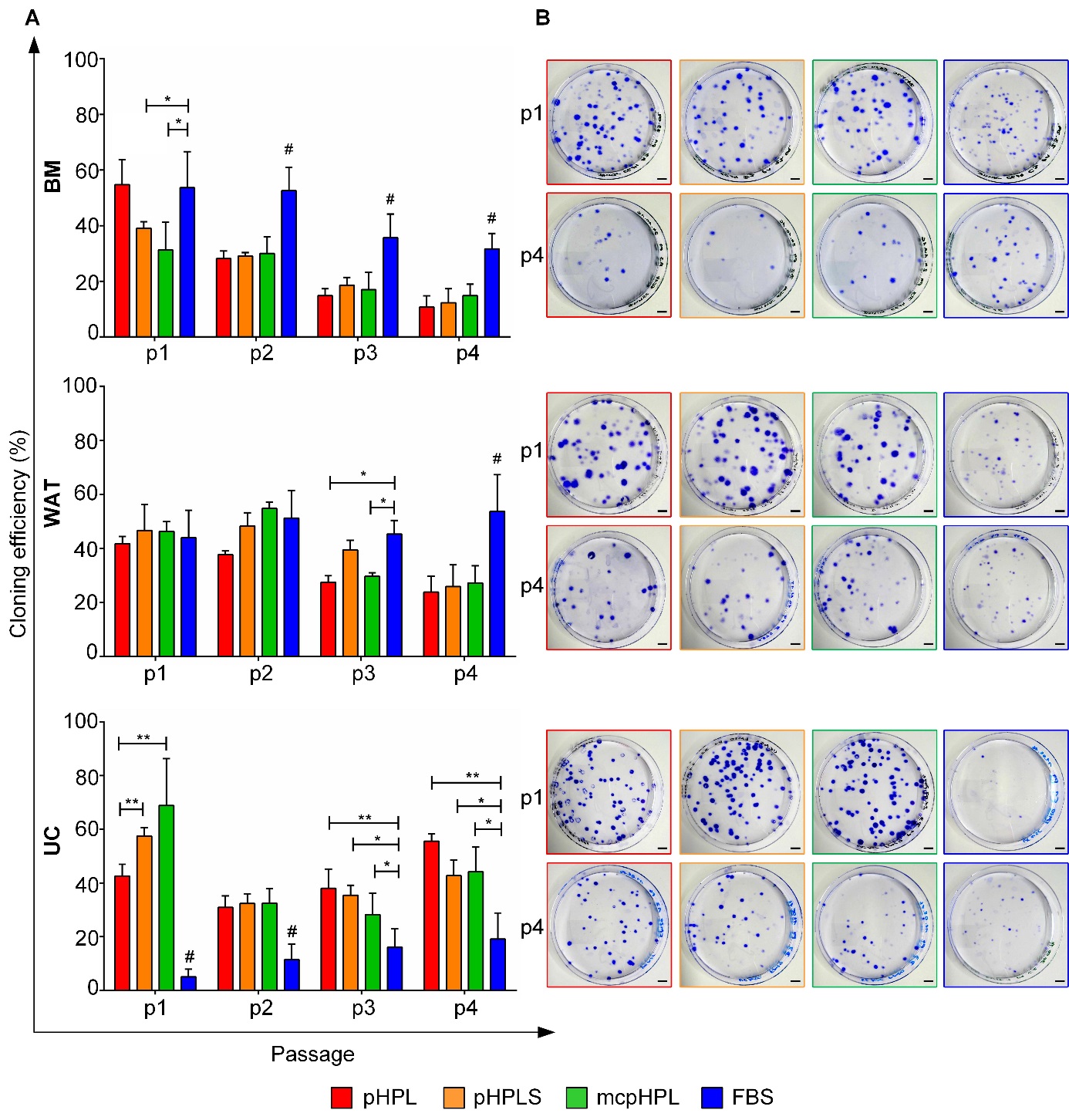


**Additional File 5: Cloning efficiency of stromal cells in different pHPL-media reveals tissue-source dependent effects**

(**A**) Cloning efficiency (%) of BM-, WAT- and UC-derived stromal cells (for each source n = 3, analyzed in triplicates) over four passages in different pHPL- and FBS-supplemented media. Data are presented as mean of triplicates ± SD (* p < 0.05, ** p < 0.01, *** p < 0.001, # p < 0.05 in comparison to all other groups).

(**B**) Photographs of colony forming units (CFUs) from one representative donor cultured in pHPL-, pHPLS-, mcpHPL- or FBS-supplemented medium are shown for passages 1 and 4. Scale bar = 1 cm.
