# Supplementary material for: Upregulation of mitotic bookmarking factors during enhanced proliferation of human stromal cells in human platelet lysate
Source: J Transl Med. 2019 Dec 30;17:432. doi: 10.1186/s12967-019-02183-0 (PMC6936143; doi:10.1186/s12967-019-02183-0)
Supplement: Supplementary file 6 — Additional file 6. Immunophenotype and in vitro osteogenic and adipogenic differentiation of stromal cells. [file 12967_2019_2183_MOESM6_ESM.docx]

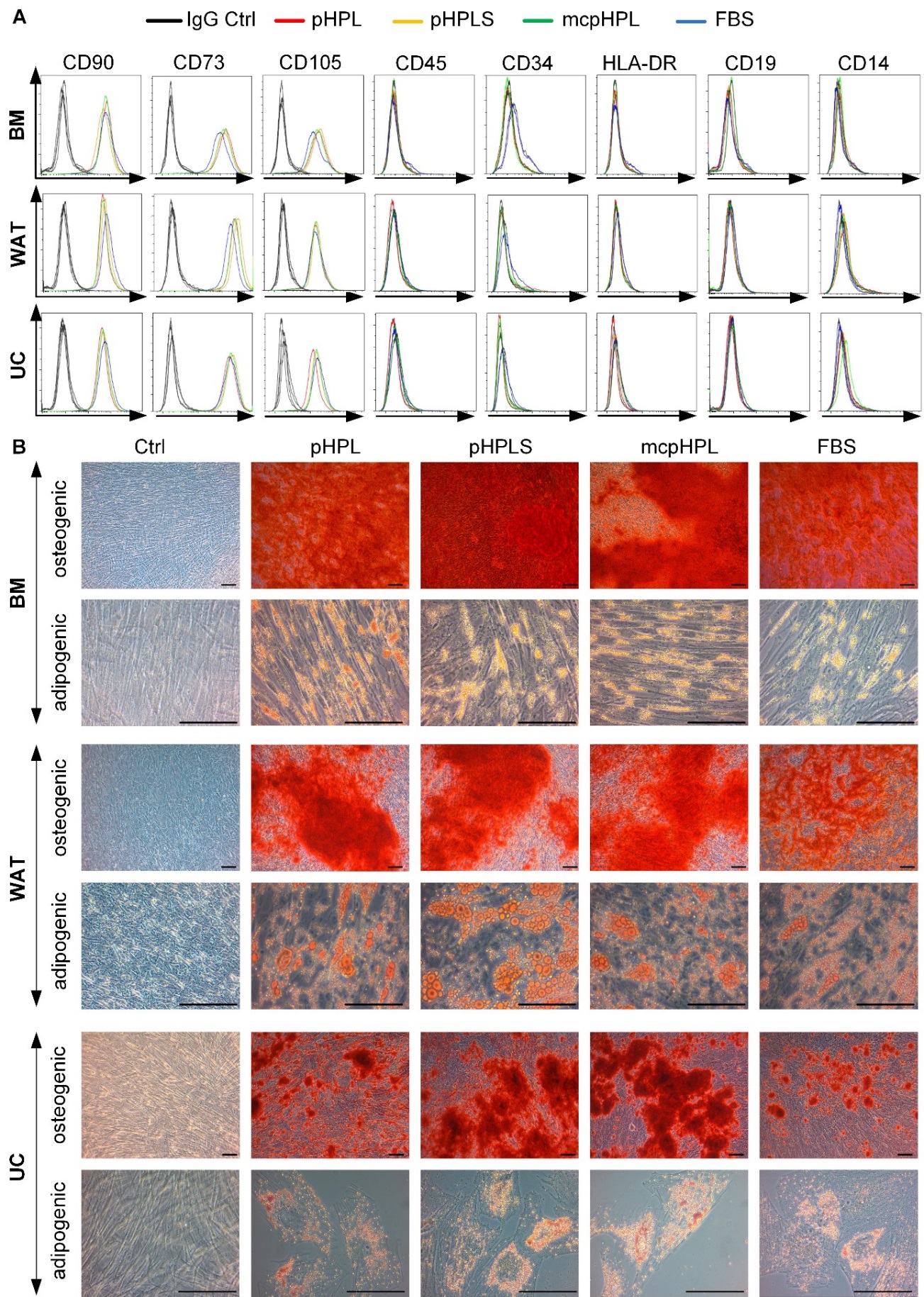


**Additional File 6: Immunophenotype and *in vitro* osteogenic and adipogenic differentiation of stromal cells**

(**A**) Flow cytometry analysis revealed the canonical immunophenotype of stromal cells isolated from bone marrow (BM), white adipose tissue (WAT) and umbilical cord (UC). Histogram plots show results of one representative stromal cell donation cultured in pHPL- (red), pHPLS- (orange), mcpHPL- (green) and FBS- (blue) supplemented medium as overlays. IgG controls are indicated in black.

(**B**) *In vitro* osteogenic and adipogenic differentiation of stromal cells of one representative donor in different culture media are displayed as indicated. Total magnification 40x for osteogenic differentiation and corresponding control (staining: Alizarin Red), 200x for adipogenic differentiation and control (staining: Sudan III), scale bar: 100 µm.
