# Supplementary material for: Upregulation of mitotic bookmarking factors during enhanced proliferation of human stromal cells in human platelet lysate
Source: J Transl Med. 2019 Dec 30;17:432. doi: 10.1186/s12967-019-02183-0 (PMC6936143; doi:10.1186/s12967-019-02183-0)
Supplement: Supplementary file 7 — Additional file 7. In vitro chondrogenic differentiation of bone marrow-derived stromal cells. [file 12967_2019_2183_MOESM7_ESM.docx]

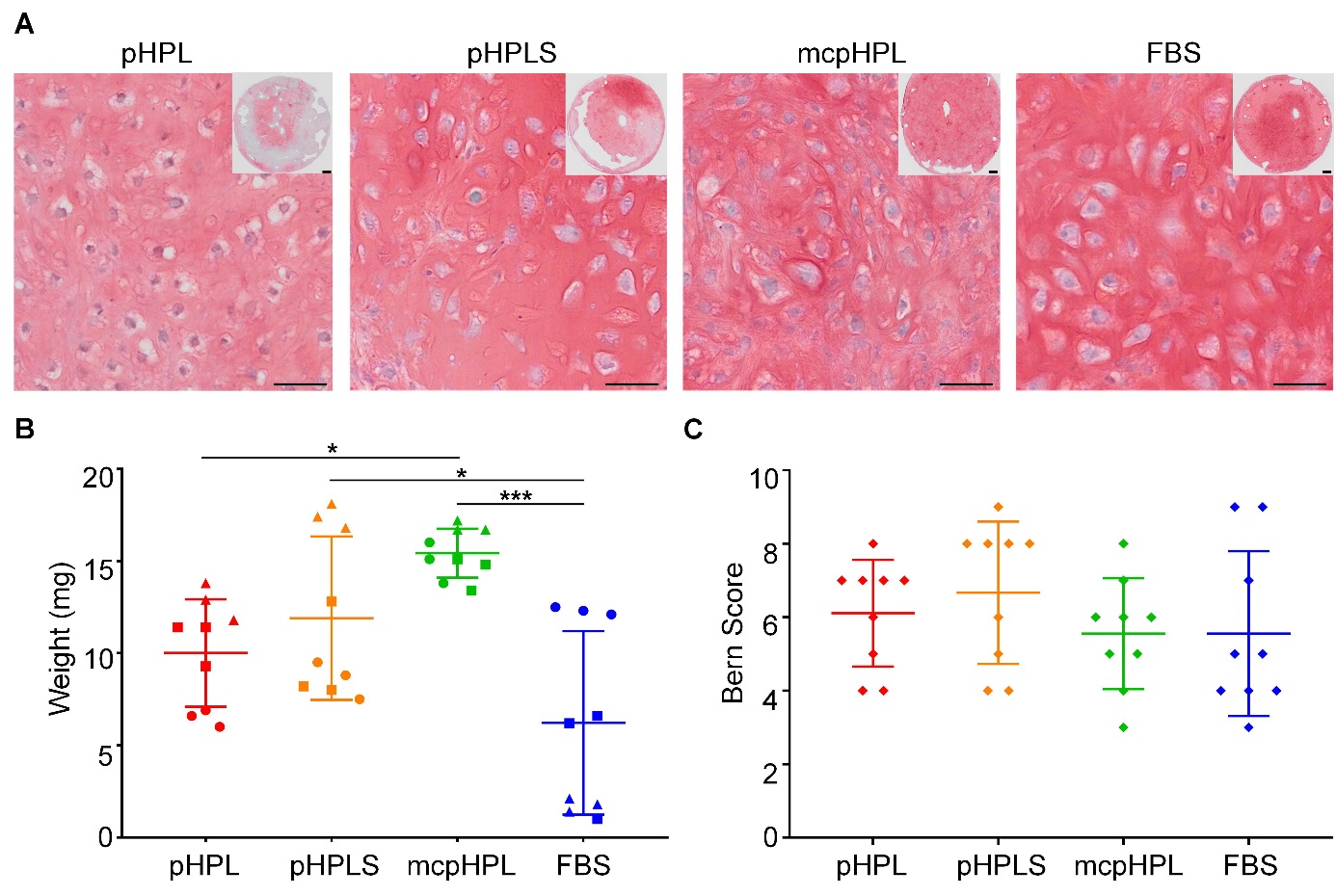


**Additional File 7: *In vitro* chondrogenic differentiation of bone marrow-derived stromal cells**

(**A**) *In vitro* 3D chondrogenic differentiation of one representative donor out of three tested for BM-derived stromal cells in different culture media is displayed as indicated. SafraninO/FastGreen staining indicates *in vitro* chondrogenesis. Scale bar: 100 µm, scale bar insert: 500 µm. (**B**) Results were evaluated by determining the weight of the 3D cartilage discs (* p < 0.05, *** p < 0.001) and (**C**) by applying a visual histological grading system (Bern Score) for the evaluation of the SafraninO/FastGreen staining of three donors.
